# Beyond the leaves: functional role of chlorophyllous stems in tomato (*Solanum lycopersicum* L.) and their impact on nitrogen balance and root development

**DOI:** 10.1186/s12870-026-08992-y

**Published:** 2026-05-19

**Authors:** Miron Gieniec, Andrzej Kornaś, Maciej Kocurek, Marzena Sujkowska-Rybkowska, Isabel Nogués, Emanuele Pallozzi, Walter Stefanoni, Zbigniew Miszalski

**Affiliations:** 1 W. Szafer Institute of Botany, Polish Academy of Sciences, Lubicz 46, Krakow, 31–512 Poland; 2 Institute of Biology and Earth Sciences, University of the National Education Commission, Podchorążych 2, Krakow, 30–084 Poland; 3 Institute of Biology, The Jan Kochanowski University, Uniwersytecka 7, Kielce, 25–406 Poland; 4 Department of Botany and Plant Physiology, Institute of Biology, Warsaw University of Life Sciences, Nowoursynowska 159, Warsaw, 02-776 Poland; 5 Institute of Research on Terrestrial Ecosystems (IRET), National Research Council of Italy (CNR), Montelibretti (RM), 00010 Italy; 6 National Biodiversity Future Center (NBFC), Palermo, 90133 Italy

**Keywords:** ^13^C and ^15^N isotope composition, Chlorophyll *a* fluorescence, Chloroplast ultrastructure, Microscopic analysis, Nitrogen management in plants, Non-foliar photosynthesis, Photochemical processes, Root biomass

## Abstract

**Background:**

Photosynthetic activity in plant stems, particularly in woody plants and shrubs, is well known and documented. Nevertheless, our knowledge about the role of photochemical processes occurring in the stems of cultivated plants and their contribution to plant growth and development, including the root system, still remains unclear. Therefore, this study aims to determine the role of cells equipped with chloroplasts and located in the stems of tomato (*Solanum lycopersicum* L.) plants and whether changes in the activity of the photochemical processes affect the development of the plant’s root system and aboveground organs.

**Results:**

The described study was conducted on tomato plants with stems covered with a doubled agrotextile (DP) that absorb 99% of incident light and on plants growing without coverage (NDP). Stem darkening led to the changes in the intensity of photochemical processes in these organs - a decrease in both the maximum and actual photochemical efficiency [F_v_/F_m_ and Y(II)], as well as an increase in energy dissipation, both controlled (NPQ – non-photochemical quenching) and uncontrolled [(Y(NO) - non-regulated energy dissipation in photosystem II] way. These changes correlate with alterations in the anatomy of the stem cross-section. Darkening led to an increase in stem surface conductance to water vapour but a decrease in stem CO_2_ efflux. The root length and dry mass of DP plants were significantly reduced compared to the roots of NDP plants. No significant differences were observed for the shoots (leaves + stems). The substantial changes in the structure of chloroplasts located in the stem cells of DP were also observed, with visible signs of ageing and disintegration of these organelles. In addition, the composition of the nitrogen forms in the soil where the plants were grown was different between DP and NDP. The soil from DP plants showed a higher total nitrogen content; however, unlike the ammonium and nitrate forms (NH_4_⁺ and NO_3_⁻), the nitrite form (NO_2_⁻) was present at a lower concentration in this substrate compared to that of the NDP plants. Darkening did not have a direct effect on the δ¹³C and δ¹⁵N composition between NDP and DP plants, nor on the carbon-to-nitrogen ratio in the leaves, roots, and stems. Nevertheless, based on the obtained results for δ¹³C, δ¹⁵N, and the nitrogen-to-carbon ratio, some general trends can be observed.

**Conclusions:**

The obtained results suggest that photochemical processes occurring in tomato stems have an important influence on the development of the tomato plant, especially on the root system. Limited light access to the stems restricts root system growth and development, but shows no visible negative effects on the above-ground parts of the tomato plants in this study. However, indirect effects on shoot growth and development cannot be excluded. Restricting light exposure to the stems likely reduces energy production in the form of energy carriers, such as ATP and NADPH, resulting in decreased efficiency of assimilate transport to the root. This disrupts the plant’s nitrogen uptake and balance, and in turn limits root system development. Moreover, it can be assumed that the chloroplasts present in tomato stems differ structurally and, as a consequence, functionally from those in leaves, which are specialised for efficient photosynthesis. Because a well-developed root system is an important factor affecting plant growth and yield, the obtained results may suggest the possibility of agronomic practices aimed at increasing the efficiency of photochemical processes and photosynthesis in the stems, and consequently improving yields.

**Supplementary Information:**

The online version contains supplementary material available at 10.1186/s12870-026-08992-y.

## Background

Photosynthesis is a fundamental biological process by which green plants convert light energy into chemical energy, leading to the synthesis of organic compounds, including carbohydrates, that are essential for plant growth and development. Leaves are the primary organs of photosynthesis, as their structures facilitate carbon dioxide uptake and efficient photosynthetic activity [1]. However, cells equipped with chloroplasts are also present in other plant organ as for example stems.

Photochemical processes occurring in stems should not be overlooked, even though they are severely limited, like in many trees, where chloroplast-containing cells are located under cork and cortical layers, which makes it difficult for the stem to absorb light [2]. However, stem photosynthesis is maintained despite low light availability due to subepidermal chloroplasts and internal CO₂ re-fixation [1]. Moreover, stem photosynthesis may play an important role in supporting plant development and growth, especially under stress conditions, i.e. drought, and may also serve compensatory functions by increasing the overall photosynthetic capacity of plants [1, 3–10].

Plant stems exhibit sophisticated mechanical properties and a highly organised anatomical structure, enabling them to perform multiple physiological and structural functions. These include the storage and transport of water and nutrients, adaptive growth, self-repair after fissures and wounds, and ensuring bending and torsional stiffness, vibration damping, and strength [11]. Certain stem tissues, such as the cortex, including chlorenchyma, are equipped with chloroplasts, suggesting that those tissues in stems can participate in photochemical processes, and potentially in carbon assimilation [12–14]. Stem photosynthesis has been extensively studied and documented in numerous woody species, including trees and shrubs, such as species of the *Populus* genus [10, 15–17], *Betula pendula* Roth [18], *Fagus sylvatica* L. (European beech) and *Quercus robur* L. (pedunculate oak) [2, 19, 20], *Salix matsudana* Koidz. (weeping willow) [14], *Prunus ilicifolia* (Nutt. ex Hook. & Arn.) Walp. (hollyleaf cherry), *Umbellularia californica* (Hook. & Arn.) Nutt. (California bay) and *Arctostaphylos manzanita* Parry (Manzanita) [21]. Photosynthetic activity in stems has also been reported in several desert plant species [22–26].

Despite evidence that green stems can contribute substantially to photochemical processes of herbaceous and crop species, their significance in these plants remains relatively understudied. This phenomenon has been studied in *Gossypium hirsutum* L. (upland cotton) in relation to seed weight [27]. Xu et al. [28] reported lower chlorophyll concentrations in the lower stem parts of *Solanum lycopersicum* L. (tomato); however, it was found that up to 4% of the total photosynthetic activity in this species can occur in the stems [29, 30]. The stems of two varieties of herbaceous plants - *Reynoutria japonica* Houtt. (Japanese knotweed) and *Helianthus tuberosus* L. (topinambur), characterised by high resistance of the epidermis, also uses the internal carbon dioxide in photosynthesis [31].

These observations support the view that photochemical processes and photosynthesis in stems, as well as in other plant organs such as fruits, seeds, petioles and floral structures, predominantly rely on carbon dioxide derived from internal respiration rather than from the atmosphere, and stems are not directly specialised to photosynthesis [32, 33]. According to Aschan and Pfanz [34], stem photosynthesis has been reported in at least 36 plant families, including species with herbaceous stems and that perform C_3_ photosynthesis. This is also supported by studies on Arabidopsis thaliana, in which photosynthetic proteins have been detected in stem tissues [35]. As was demonstrated by Hibberd and Quick [36], the celery and tobacco cells around the vascular bundles in the stems and petioles exhibit C_4_-like photosynthesis, although the remaining parts of the plant conduct standard C_3_ photosynthesis. In such plants, roots supply a four-carbon organic compound – malate - while C_4_-specific enzymes in stems catalyse CO_2_ release in proximity to the Calvin cycle. This mechanism creates a form of “metabolic loop”, whereby CO_2_ derived from root respiration is reassimilated in stems and subsequently converted into sugars that are transported back to the roots, thereby supporting the development of these organs. Similar observations have been reported in woody species such as *Pinus sylvestris* L. (Scots pine) [37], *Clusia minor* L. [38], as well as in the petioles and stems of the *Cucumis sativus* L. (cucumber) crop plant [39].

The benefits of photochemical processes taking place in plant stems are not restricted only to local tissues and roots. In experiments involving stem darkening, the biomass of buds in young plants of *P. ilicifolia*, *U. californica*, *A. manzanita*, and *S. matsudana* was significantly higher when stems exhibited greater photosynthetic activity compared with darkened conditions [14, 21].

Investigation of the role of photochemical processes occurring in stems is particularly important in crop plants, whose stems have a different anatomical structure from that of tree trunks. Consequently, the absorption of light and CO_2_ is often less restricted than in woody species. Moreover, understanding the contribution of these processes to the development of individual plant organs may provide valuable insights into factors influencing plant growth, yield, and tolerance to unfavourable environmental conditions.

One of the most important crop species with global production reaching approximately 195 million tons in the world in 2023 is the tomato (*S. lycopersicum*) [40]. Tomato fruits constitute an important source of nutrients, including vitamins A and C, as well as potassium [41]. Furthermore, due to its traits – relatively small genome and short life cycle, tomato is considered a suitable model species for studying developmental and physiological processes occurring in plants [42]. Yet, little is known about whether tomato stems contribute functionally to plant carbon balance or nutrient uptake.

To date, most studies on photochemical processes in stems have focused on woody plants and shrubs, whereas considerably less information is available for herbaceous species, particularly crop plants such as tomato. However, despite extensive information on woody species, the functional significance of stem photochemistry in herbaceous crops remains unclear, especially with regard to root development and nitrogen uptake. Therefore, in this study, we aimed to investigate potential changes in photochemical processes in chloroplasts of stem cells in ‘Red Pear’ tomato plants following partial stem darkening using an agrotextile. We hypothesised that limited light availability to tomato stems would reduce biomass accumulation and plant development. We assumed that stem darkening would disrupt photochemical processes and affect the development of above- and below-ground systems as a result of modifications in chloroplast ultrastructure and carbon and nitrogen metabolism.

In this study, we sought to determine the role of cells located in the stem that contain chloroplasts and whether photochemical processes taking place within stems regulate the transport of photoassimilates, affecting the development of the plant’s root system and aboveground organs. To our knowledge, no previous study has linked stem darkening to alterations in soil nitrogen uptake patterns in tomato.

To address the mentioned goals, we measured the dry mass of roots and aboveground plant parts. The effects of light limitation were evaluated by measuring chlorophyll *a* (Chl*a*) fluorescence in stems, pith and leaves, making it possible to analyse whether changes occurring in stems affect the overall efficiency of the photosynthetic mechanism in leaves. To examine the role of stems in carbon and nitrogen metabolism, the redistribution of resources between plant organs, as well as dependencies between the stem, its photochemical activity and nitrogen and carbon management in plants, we measured the content of nitrogen forms in the soil and C: N ratio and ^13^C and ^15^N isotope composition in roots, stems and leaves. Additionally, potential differences in the anatomy of the cross-sections of non-darkened and darkened plants, as well as the ultrastructure of chloroplasts, were examined using fluorescence and electron microscopy techniques, respectively.

In this study, the term “stem photosynthesis” refers to light-dependent photochemical processes occurring in stem chloroplasts.

## Results

### Stem darkening reduces root growth

**Fig. 1:**
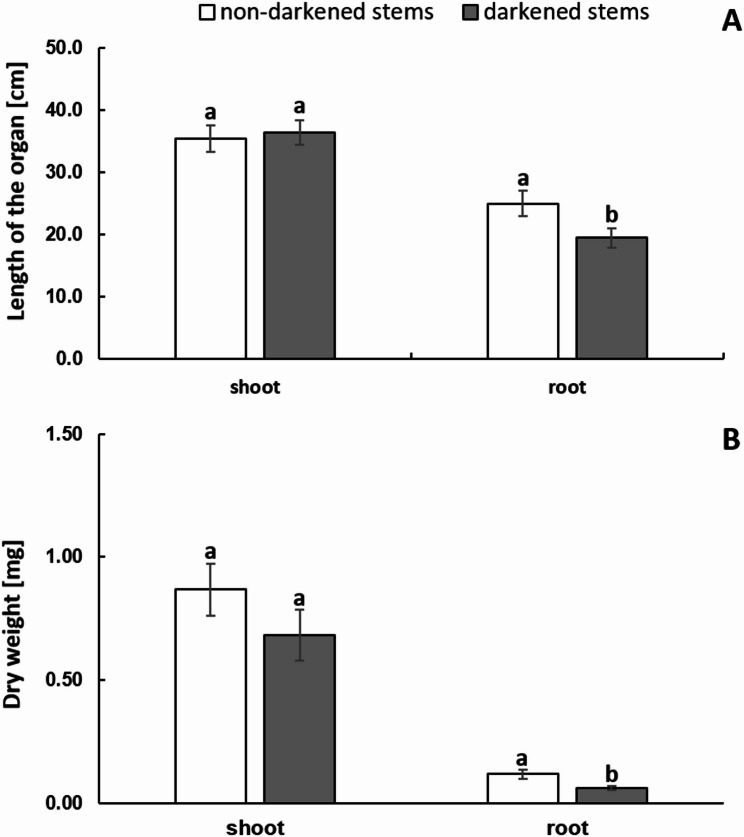
Average length (**A**) and dry mass (**B**) of organs – shoots (stems+leaves) and roots of 8-week-old ‘Red Pear’ tomato (*Solanum lycopersicum* L.) plants with non-darkened (white background) and darkened (grey background) stems. The means followed by the same letters do not differ significantly according to the *t*-test or Mann-Whitney test at *p* ≤ 0.05. Analyses were conducted separately for each organ (*n* = 13). Whiskers represent standard deviation values

A comparison of stem and root system morphology showed that 6-week stem darkening significantly reduced root length and dry mass in *S. lycopersicum* variety - ‘Red Pear’. However, no significant differences were observed in shoot (stem+leaves) dry weight or length (Fig. 1A-B). Roots of plants with agrotextile-covered stems (darkened plants - DP) were significantly shorter (19.5 ± 5.32 cm) compared to those of uncovered plants (non-darkened plants - NDP) (25.0 ± 7.33 cm). Likewise, the dry weight of DP roots was lower (0.061 ± 0.030 mg) than that of NDP roots (0.12 ± 0.067 mg). The results obtained for shoots (stem + leaves) did not show significant differences. Moreover, the mean stem length of the DP plants was slightly higher (36.38 ± 6.98 cm) compared with NDP plants (35.46 ± 7.74 cm).

### Stem darkening reduces PSII efficiency in stems but enhances photochemical activity in leaves

**Fig. 2:**
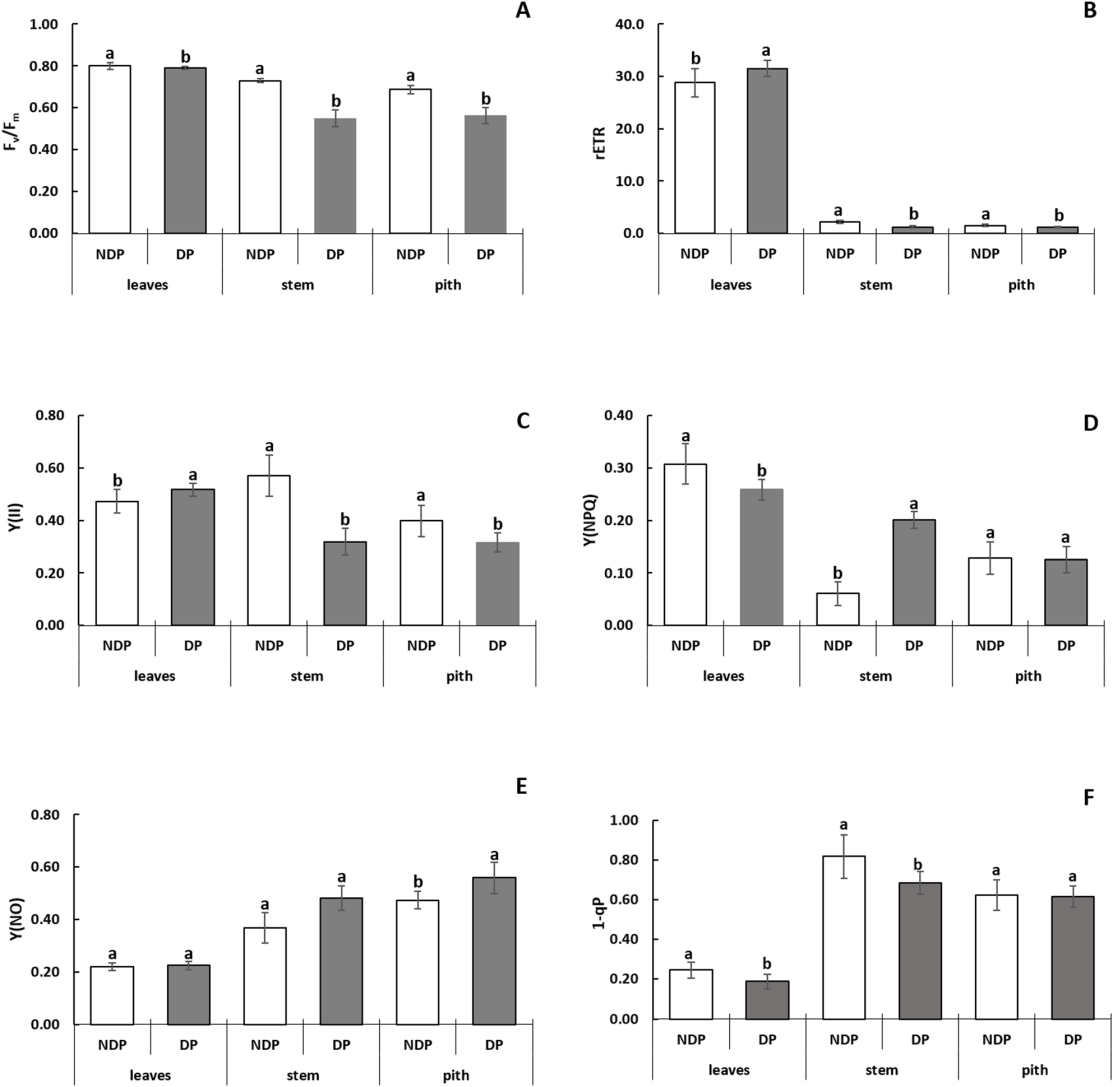
Photochemical parameters of leaves, stems, and piths of 8-week-old ‘Red Pear’ tomato (*Solanum lycopersicum* L.) plants with non-darkened (white background; NDP) and darkened (grey background; DP) stems: maximum quantum yield of PSII – F_v_/F_m_ (**A**), relative electron transport rate – rETR (**B**), effective quantum yield of photosystem II (PSII) – Y(II) (**C**), yield of regulated non-photochemical quenching – Y(NPQ) (**D**), yield of non-regulated non-photochemical quenching – Y(NO) (**E**) and proportion of closed PSII reaction centres (1−qP) (**F**). All parameters are dimensionless. In the graph, the term ‘stem’ refers to all stem tissues excluding the pith. Raw F_0_ and F_m_ values used for F_v_/F_m_ calculation are provided in Supplementary Table S1. The means followed by the same letters do not differ significantly according to the *t*-test or Mann-Whitney test at *p* ≤ 0.05. Analyses were conducted separately for each organ (*n* = 10 for leaves and 8 for stem and pith). Whiskers represent standard deviation values. Measurements were performed under actinic light at 145 µmol photons m⁻² s⁻¹ for leaves and 9 µmol photons m⁻² s⁻¹ for stems

For Chl*a* fluorescence analyses, the pith was considered as a separate tissue, while the term ‘stem’ refers to the remaining stem tissues excluding the pith. Although no differences were observed in stem morphology between NDP and DP tomato plants, Chl*a* fluorescence analyses showed differences in photosystem II (PSII) parameters for leaves, stems, and pith separately (Fig. 2). Moreover, both the direction and magnitude of these changes consistently differed between the experimental variants (NDP and DP) for stems and leaves across the measured parameters.

The maximum PSII photochemical efficiency parameter (maximum quantum yield of photosystem II - F_v_/F_m_) differed significantly (Fig. 2A), and the values were lower in the leaves, stems and pith of DP compared to NDP plants.

Relative electron transport rate (rETR) was significantly higher in DP leaves compared to NDP leaves. In stems and pith, rETR was significantly reduced in DP plants (Fig. 2B). The effective quantum yield of PSII [Y(II)] was higher in DP leaves compared to NDP leaves, whereas the opposite trend was observed in stems and pith, where darkening induced a decrease in this parameter (Fig. 2C).

Distinct patterns were also observed for non-photochemical quenching parameters, Y(NPQ) and passive energy dissipation, Y(NO). Both parameters were higher in DP stems than in NDP stems. In contrast, Y(NPQ) was higher in NDP leaves than in DP leaves, whereas Y(NO) showed no significant differences in leaves (Fig. 2D-E). The proportion of closed PSII reaction centres (1 − qP) was higher in NDP leaves than in DP leaves, and a similar pattern was observed in stems (Fig. 2F).

### Stem darkening alters stem anatomy

**Fig. 3:**
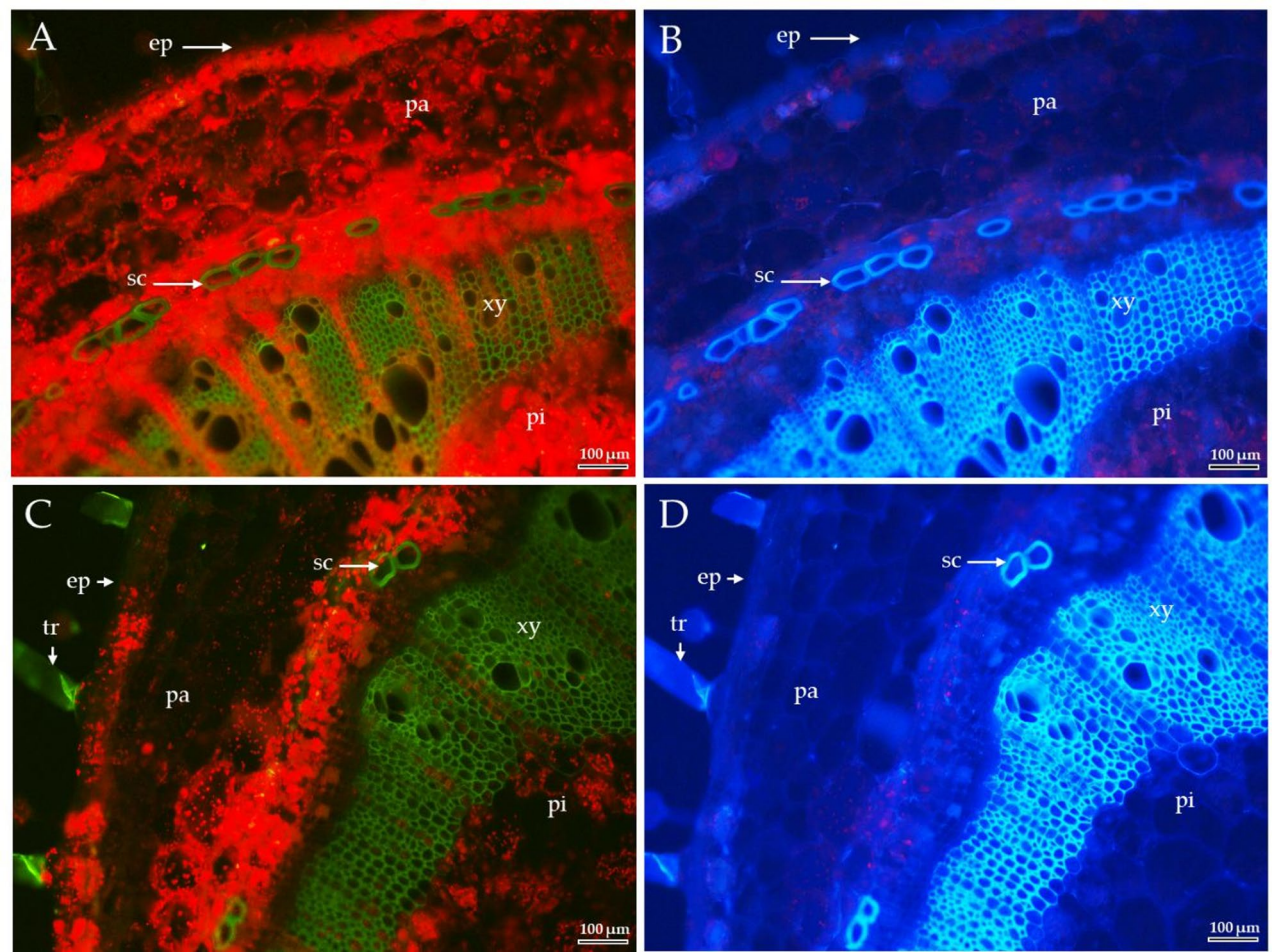
Photography of the cross-section of the stem of the 8-week-old ‘Red Pear’ tomato (*Solanum lycopersicum* L.) plants with non-darkened (**A**, **B**) and darkened stems (**C**, **D**) observed by epifluorescence microscopy. The imaging was conducted once for experimental variant. Red and green colours correspond to autofluorescence of chlorophyll and cell walls, respectively and blue colour corresponds to fluorescence of the lignin. ep – epidermis; p.a. – parenchyma; pi – pith; sc – sclereids; tr – trichome; xy – xylem. Scale bar: 100 μm

After removal of the agrotextile, the stems of DP plants appeared pale green, indicating a partially etiolated phenotype. Microscopic analysis of stem cross-sections revealed pronounced differences in stem anatomy between DP and NDP. These differences were also associated with distinct patterns of chlorophyll-related fluorescence (Fig. 3). Chlorophyll content was not directly measured; instead, qualitative observations of red autofluorescence were used to infer its distribution. In NDP stems, red autofluorescence of Chl*a* was observed throughout all stem tissues, including the epidermis, cortex parenchyma, and pith (Fig. 3A). In contrast, DP stems exhibited a lower chlorophyll signal, particularly in the pith and parenchyma regions (Fig. 3C). The sclerenchymatous tissue was reduced in NDP stems. In Fig. 3B and D, the presence of lignin is shown.

### Stem darkening increases stem surface conductance to water vapour and reduces CO_2_ efflux

**Fig. 4:**
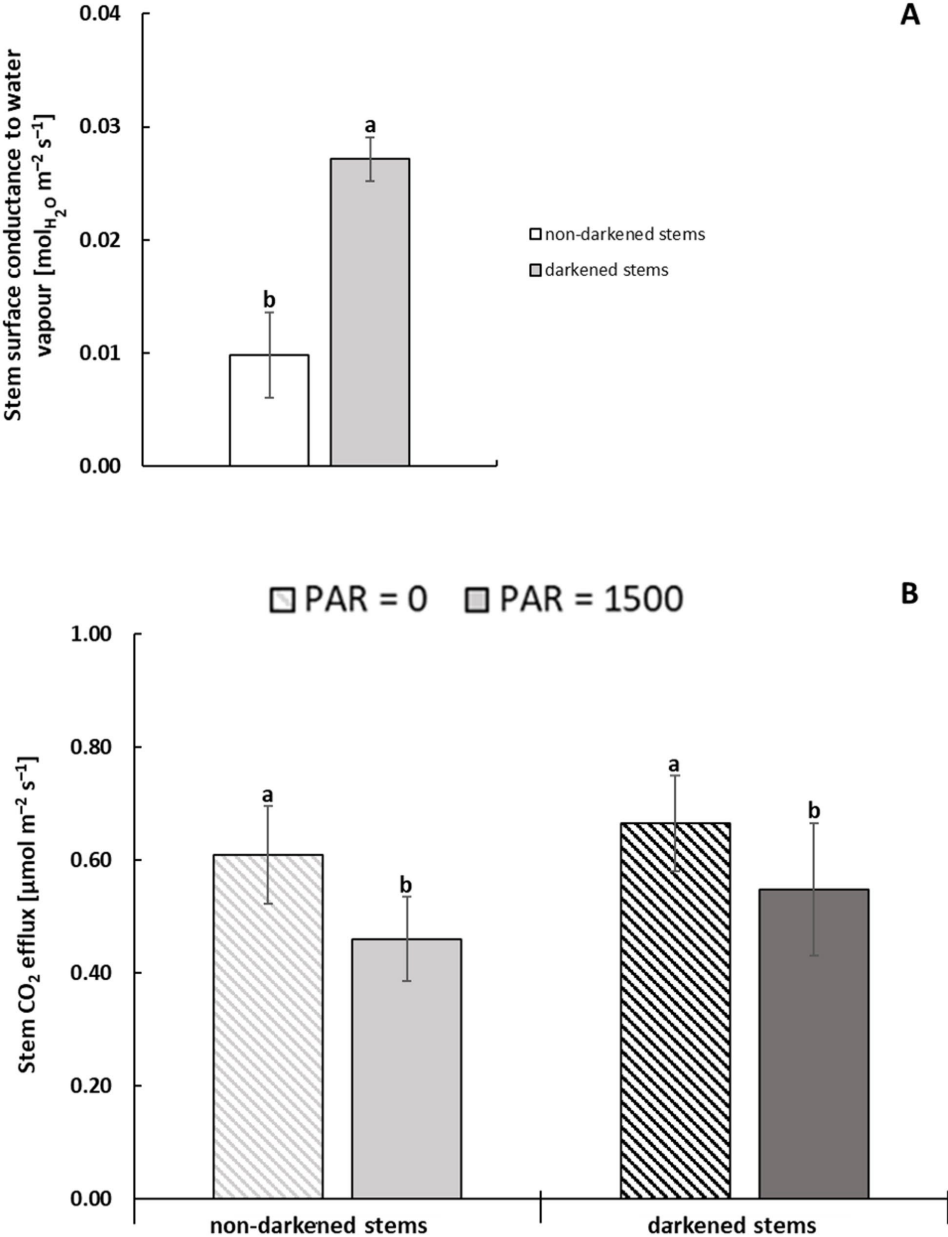
Stem gas exchange parameters of 8-week-old ‘Red Pear’ tomato (*Solanum lycopersicum* L.) plants: stem surface conductance for water vapour (mol H_2_O m⁻^2^ s⁻^1^) for plants with non-darkened (white background) and darkened (grey background) stems (**A**) and carbon dioxide efflux (µmol CO_2_ m⁻^2^ s⁻^1^) for plants with non-darkened and darkened stems (**B**). Measurements were performed at two levels of the photosynthetically active radiation (PAR): darkness (0 µmol m⁻^2^ s⁻^1^) and high light intensity (1500 µmol m⁻^2^ s⁻^1^) (**B**). The means followed by the same letters do not differ significantly according to the *t*-test at *p* ≤ 0.05. Analyses were conducted separately for each variant (darkened and non-darkened stems) (*n* = 4)

Significant differences were observed in the value of stem surface conductance to water vapour measured as the H_2_O efflux, which increased in DP stems compared to NDP stems, with mean values rising from 0.0098 mol H₂O m⁻² s⁻¹ in NDP to 0.027 mol H₂O m⁻² s⁻¹ in DP (Fig. 4A). A significant reduction in stem CO₂ efflux was observed under 1500 µmol m⁻² s⁻¹ photosynthetically active radiation (PAR) comparing to darkness (0 µmol m⁻² s⁻¹) in both NDP and DP stems indicating re-assimilation of internal CO_2_. The mean stem CO₂ efflux ranged from 0.46 to 0.64 µmol CO₂ m⁻² s⁻¹ (Fig. 4B). No significant differences were found in stem CO₂ efflux in the dark and light between DP and NDP stems.

### Stem darkening reduces nitrogen uptake, but does not significantly affect carbon and nitrogen isotope composition

**Fig. 5:**
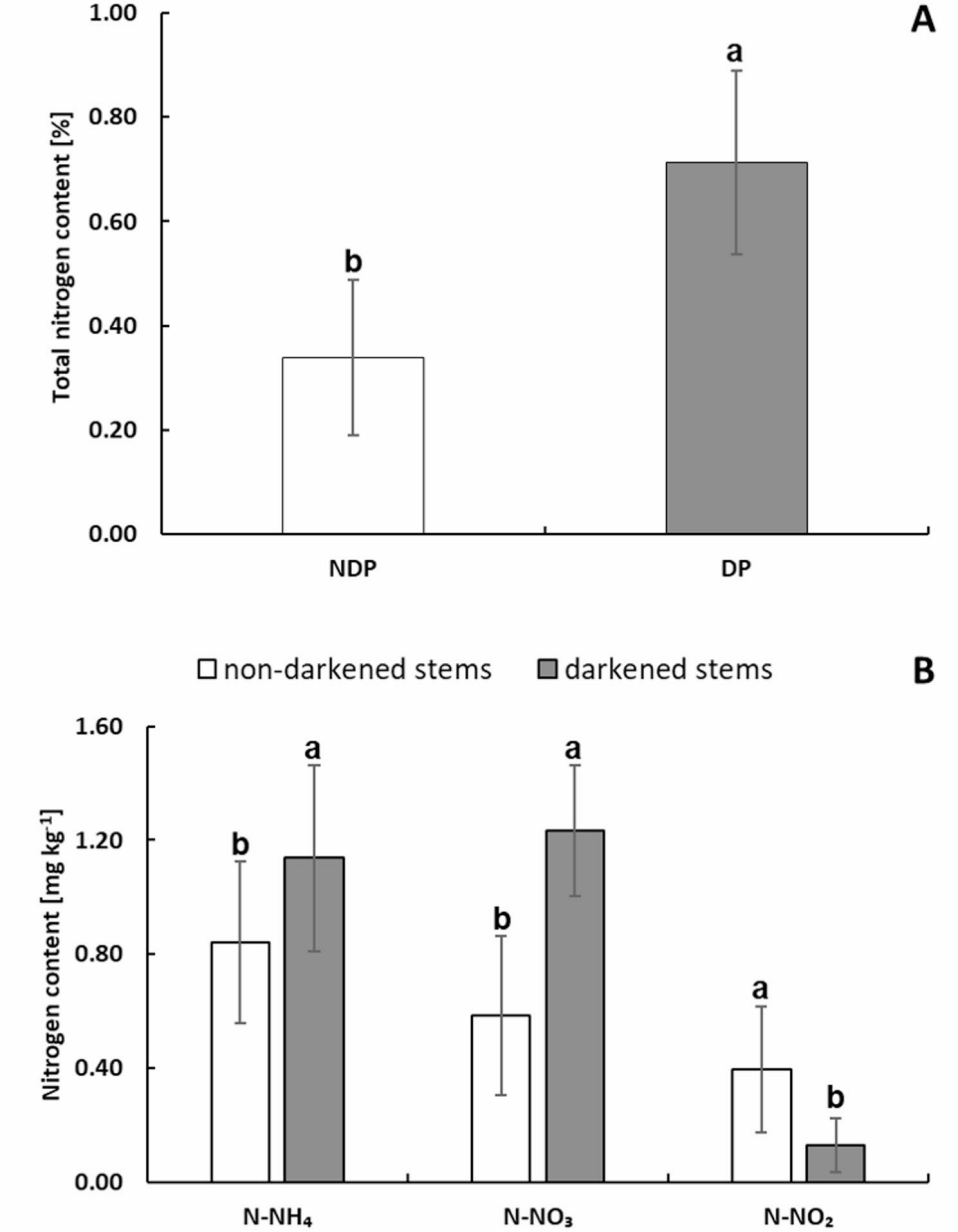
The total nitrogen content (**A**) and content of various forms of nitrogen in the soil (**B**) on which 8-week-old ‘Red Pear’ tomato (*Solanum lycopersicum* L.) plants with non-darkened (NDP, white background) and darkened (DP, grey background) stems (DP, grey background) were cultivated. The means followed by the same letters do not differ significantly according to the *t*-test or Mann-Whitney test at *p* ≤ 0.05 between the two treatments, determined separately for each nitrogen form (*n* = 7 for total nitrogen and N-NO_3_ form content and *n* = 6 for N-NH_4_ and N-NO_2_ form content)

The total nitrogen content was significantly higher in the soil where DP plants were grown (0.71% vs. 0.34% for NDP) (Fig. 5A). A similar trend was observed for N-NO_3_ form (1.23 vs. 0.58 mg kg^−1^ of soil) and for ammonium nitrogen form (N-NH_4_^+^) (1.14 vs. 0.84 mg kg⁻^1^ of soil). Higher nitrogen content in the N-NO_2_ form was noted for NDP (0.40 vs. 0.13 mg kg⁻^1^ of soil) (Fig. 5B).

**Fig. 6:**
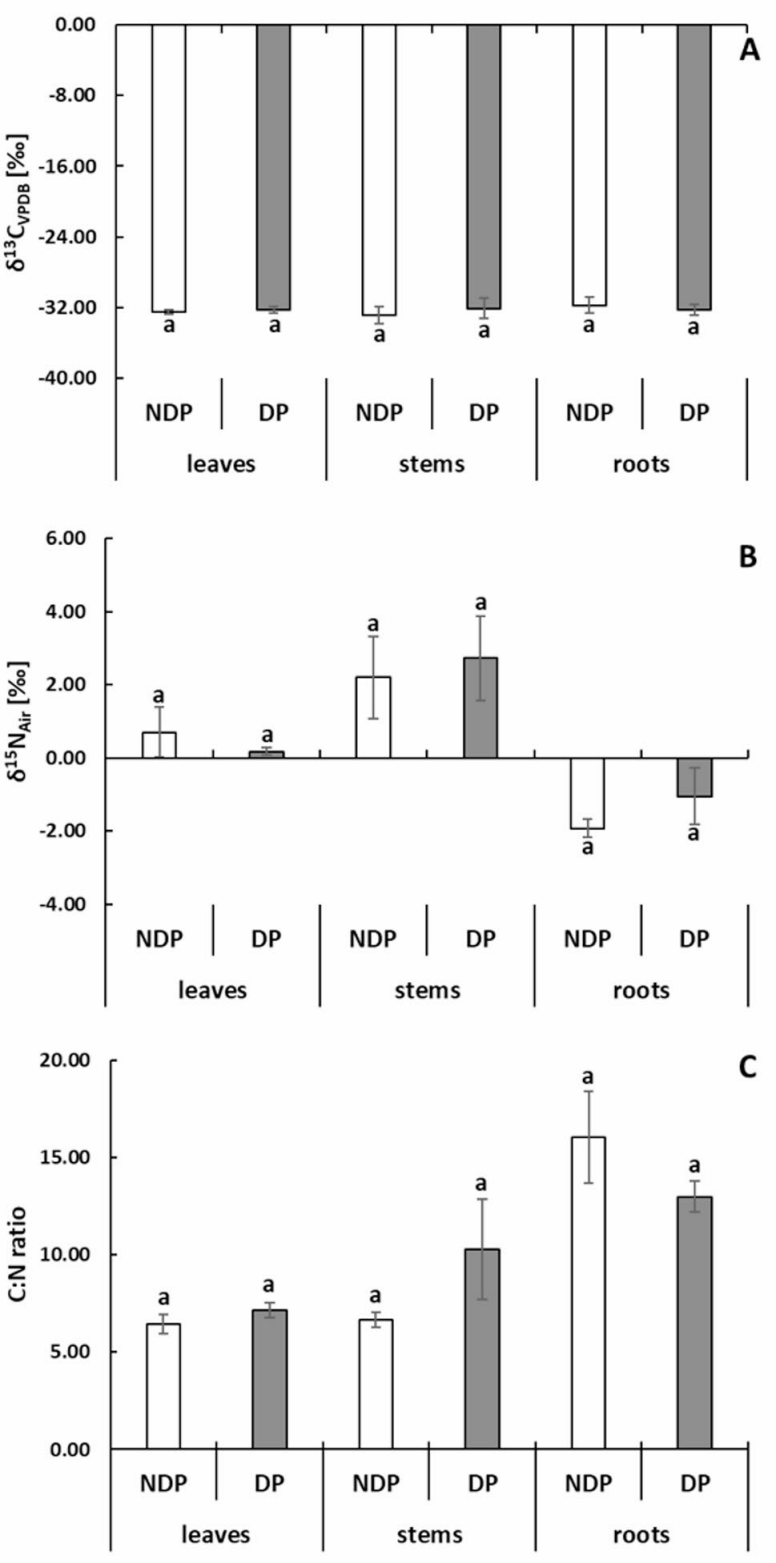
^13^C isotope composition (δ^13^C) (**A**), ^15^N isotope composition (δ^15^N) (**B**) and the ratio of total content carbon and nitrogen (C: N) (**C**) in organs (leaves, roots and stems) of 8-week-old ‘Red Pear’ tomato (*Solanum lycopersicum* L.) plants with non-darkened (NDP, white background) and darkened stems (DP, grey background). The means followed by the same letters do not differ significantly according to the *t*-test or Mann-Whitney test at *p* ≤ 0.05. Analyses were conducted separately for each organ and each parameter (*n* = 4).

The differences in the composition of carbon isotope (δ^13^C) were not statistically significant. However, slightly lower δ^13^C values were obtained for leaves and roots of DP than for NDP, but higher for stems of NDP than for DP. Within the experimental variant (NDP or DP), the lowest δ^13^C values were revealed in leaves and the highest in the roots. Less negative δ¹³C values indicate a higher ^13^C content (Fig. 6A). Similarly, the differences in the composition of nitrogen isotope (δ^15^N) were not statistically significant. Slightly higher values were measured for leaves of NDP and for stems and roots of DP. Within the experimental variant (NDP or DP), the lowest and negative δ^15^N values were observed for roots and the highest (positive values) for stems (Fig. 6B). A higher value of the C: N ratio was measured for leaves and stems of DP and for roots of NDP. Within the experimental variant (NDP or DP), the lowest ratio was observed in leaves and the highest in roots (Fig. 6C). These differences were also not statistically significant.

### Stem darkening leads to chloroplast degradation

**Fig. 7:**
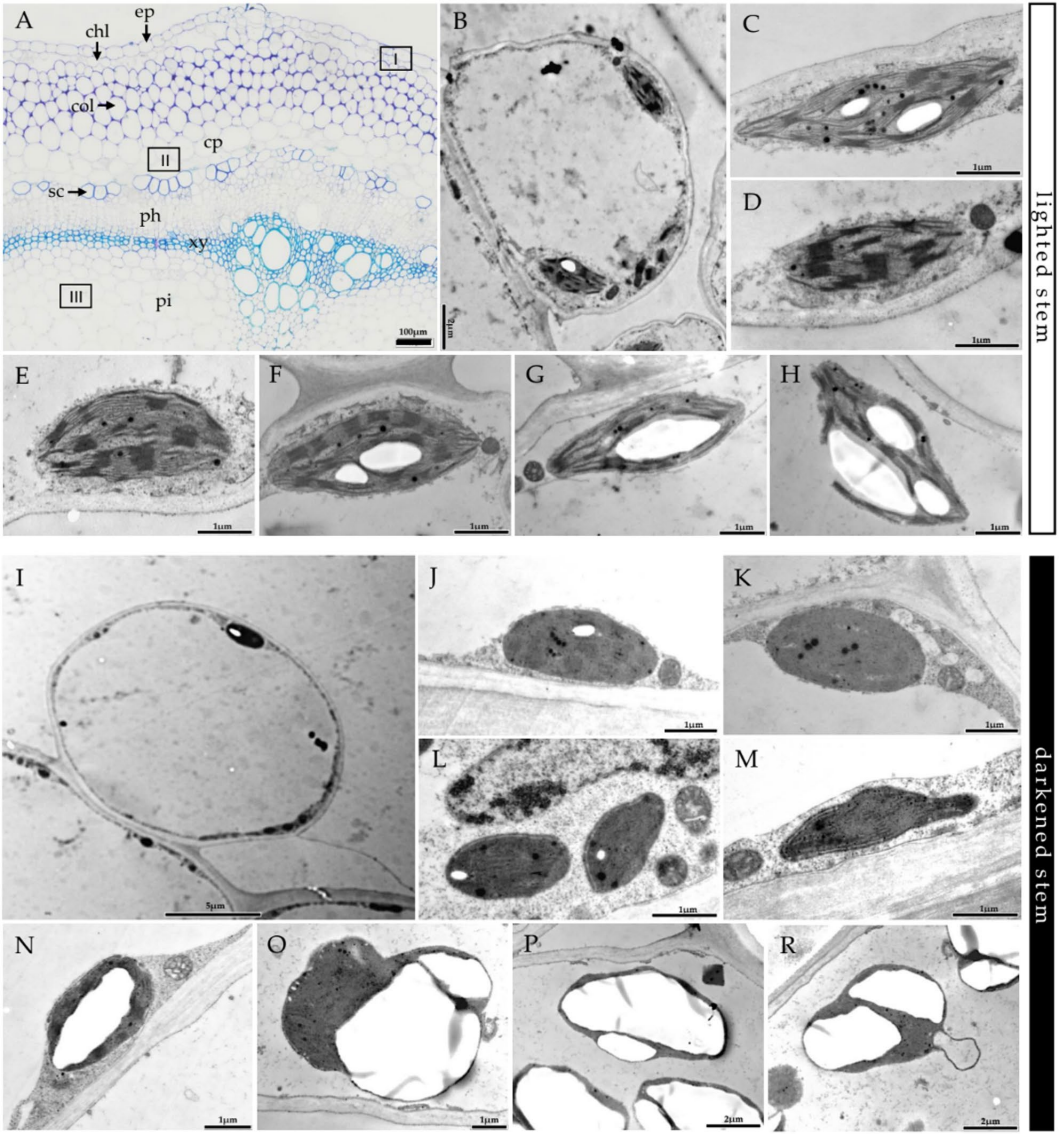
Electron microscope images of chloroplasts in stems of 8-week-old ‘Red Pear’ tomato (*Solanum lycopersicum* L.) plants with non-darkened (**A**-**H**) and darkened stems (**I**-**R**). The images originate from a single biological replicate of the experimental variant. **A**: I – chlorenchyma; II – cortical parenchyma; III – pith parenchyma; **B**−**D**: Chlorenchyma (I); chloroplasts with well-developed grana, small plastoglobules, and fine starch grains; Fig. E−F: Cortical parenchyma (II); chloroplast with wide grana, without starch and with starch, respectively; **G**−**H**: Pith parenchyma; chloroplasts showing signs of aging, still with grana system, large starch grains disrupting surrounding membranes, and numerous plastoglobules. **I**−**K**: Chloroplasts with disrupted thylakoid structure, large plastoglobules, small starch grains or their absence. **L**: Transitional stage between chloroplast and etioplast; **M**: Etioplast; **N**: Cortical parenchyma (II); chloroplast with a large starch grain showing visible granal structure; **O**−**R**: Medullary parenchyma; ageing chloroplasts, shape deformations including membrane protrusions, very large starch grains, numerous plastoglobules, and loss of grana system; chl – chlorenchyma; col – collenchyma; cp. – cortex parenchyma; ep – epidermis; ph – phloem; pi – pith; sc – sclereids; xy – xylem

Ultrastructural analysis of chloroplasts in stems of 8-week-old tomato plants revealed differences between stems of NDP and DP (Fig. 7). Chloroplasts present in stems of NDP were well developed with wide grana (Fig. 7C-E) and small plastoglobules (black dots in Fig. 7C-H). In some chloroplasts, single (Fig. 7G), double (Fig. 7C and F) or more starch grains (Fig. 7H) were visible. Chloroplasts were present in chlorenchyma (Fig. 7B-D), cortical parenchyma (Fig. 7E-F) and pith parenchyma (Fig. 7G-H). In the pith parenchyma, chloroplasts showed large starch grains and the presence of plastoglobules. In DP stems, disruption symptoms are visible (Fig. 7I-R). Chloroplasts contain damaged thylakoid structures, small starch grains or their absence (Fig. 7I-K). In stems, also organelles in the transitional stage between chloroplasts and etioplasts (Fig. 7L), etioplasts (Fig. 7M), and structures with deformations, including membrane protrusions, large starch grains, numerous plastoglobules, and loss of the grana system, were observed. The etioplast and amyloplast formation was visible.

## Discussion

Photochemical processes occur in stems as well as in other non-foliar tissues, such as flowers, fruits [1, 34]. However, it remains unclear how reduced light availability in stem tissues affects the efficiency of these processes and, consequently, the growth and development of individual plant organs, particularly roots.

### Effect of stem darkening on biomass partitioning and growth of tomato plants

The darkening of tomato stems affected biomass allocation and resulted in a statistically significant reduction in root dry mass and length. These results indicate a shift in the assimilate distribution among plant organs.

Similar patterns have been reported in other plant species. In *Rosa chinensis* [43], stem darkening reduced total biomass and increased allocation to shoots. In young, woody plants, limitation of light access to stems reduced trunk radial growth and biomass, particularly in defoliated trees [21]. Likewise, in *C. minor*, a significant reduction in root biomass and shoot/root weight ratio index has been observed [38].

Overall, these studies suggest a similar physiological response to stem darkening.

### Chlorophyll fluorescence and PSII photochemistry under stem darkening

Because different actinic light intensities were used for leaves and stems, comparisons between these organs should be interpreted qualitatively rather than quantitatively.

Chlorophyll fluorescence parameters such as Fv/Fm, rETR, and Y(II) are widely used to assess PSII performance. Fv/Fm reflects the maximum potential efficiency of PSII, whereas rETR and Y(II) describe the actual electron transport efficiency and light utilisation under given conditions [44–46]. It should be noted that rETR values represent relative estimates of electron transport derived from chlorophyll fluorescence measurements and do not reflect absolute photosynthetic electron flux. However, this aspect was not directly assessed in the present study.

In this study, Fv/Fm values in leaves of NDP and DP plants, as well as in the stem and pith of NDP plants, ranged from 0.69 to 0.8, indicating no severe PSII damage. In contrast, DP stems and pith showed reduced Fv/Fm (~ 0.56), suggesting decreased PSII efficiency under light limitation.

A different pattern was observed for rETR and Y(II). In DP, both parameters were higher in leaves but lower in the stem and pith, indicating enhanced photochemical processes in leaves of DP plants [46, 47].

Changes in PSII efficiency were accompanied by alterations in energy dissipation processes. In DP stems, reduced Fv/Fm may be associated with changes in PSII photochemical efficiency, together with increased energy dissipation via regulated [Y(NPQ)] and non-regulated [Y(NO)] pathways. However, the interpretation of Y(NO) as a measure of non-regulated energy dissipation remains debated, and fluorescence-derived parameters may not fully reflect the underlying physical processes in PSII [48].

The lower 1 − qP observed in DP stems compared with NDP may reflect differences in growth light conditions before measurements. However, 1 − qP can be influenced by multiple factors, including PSI activity, and should therefore be interpreted with caution [48–51].

Taken together, these results indicate that limited light availability reduced PSII photochemical activity in stems, whereas leaves exhibit increased photochemical performance, which may contribute to carbon assimilation under light limitation. These local changes in stem photochemistry may have systemic consequences, potentially through altered assimilate transport, thereby contributing to the maintenance of whole-plant carbon balance under light limitation.

### Spatial distribution of chlorophyll in the stem tissues under darkening

The reduction of chlorophyll content in DP was clearly visible in the stem cross-section, especially in the pith and parenchyma.

In NDP, a higher chlorophyll concentration was observed in the stem tissues excluding pith and parenchyma, and a similar pattern has been reported in many C_3_ trees [51].

In several CAM (Crassulacean Acid Metabolism) and xerophytic plants, chlorophyll is predominantly localised in the peripheral stem tissues, including bark and cortex regions, as reported for *C. multiflora* and *C. rosea* Jacq. [13], as well as in various desert plant species, including succulent and cactus stems [52, 53]. Chlorophyll presence was also identified in the cortex and pith of *C. minor* stem [38].

These observations indicate a different scheme of chlorophyll distribution across the plant species, probably as an adaptation to differences in light availability resulting, among others, from anatomical variation between plants.

A somewhat similar functional organisation has been proposed for photosynthetically competent cells of the midrib and the interveinal lamina in leaves. It was also found that the midrib cells may be involved at least partly in the supply of CO_2_ for carboxylation [54].

### Effect of stem darkening on CO₂ exchange and stem surface conductance to water vapour

In the examined stems, stem surface conductance to water vapour was higher under darkened conditions, while CO_2_ efflux increased in darkness compared to 1500 µmol m⁻^2^ s⁻ ^1^ PAR, and reached positive values, indicating net CO_2_ release to the environment. This suggests that probably respiratory CO₂ production exceeded its fixation.

However, the observed differences between darkness and 1500 µmol m⁻^2^ s⁻^1^ PAR mean that light is required to sustain photochemical activity in stems, confirming the presence of a functional photosynthetically active apparatus regardless of experimental variant (NDP or DP). This, in turn, may indicate that stem photochemistry may contribute, along with leaves, to maintain carbon balance in plants.

The results of stem surface conductance to water vapour are in contradiction to the results obtained mainly from trees [38, 55]. Previous studies have shown that the bark conductance for water and CO_2_ varies between tree species [56], with reported ranges of 0.9 to 27 mmol m⁻^2^ s⁻^1^ for water conductance and 0.2–4 µmol m⁻^2^ s⁻^1^[17, 18, 56, 57]. In the present study, the stem conductance of DP achieved ~ 27 mmol m⁻^2^ s⁻^1^, whereas the CO_2_ efflux did not exceed 0.8 µmol m⁻^2^ s⁻^1^.

### Ultrastructural changes in chloroplasts

All these results are supported by an analysis of cell ultrastructure. The presence of various plastid types, such as etioplasts, chloroplasts containing starch grains of various sizes, plastoglobules, and gerontoplasts (ageing chloroplasts), indicates that chloroplast transformation under stem darkening is not uniform.

The presence of etioplasts may suggest limited possibilities for the formation of new chloroplasts. Additionally, the increased number of plastoglobules in DP stems can reflect the senescence process of chloroplasts. Furthermore, the presence of starch grains of different sizes reflects the varying levels of carbohydrate accumulation and synthesis [58–60] and can be a symptom of transformation into amyloplasts and probably the start of root formation.

Overall, these ultrastructure changes indicate that stem darkening led to chloroplast reorganisation and potentially affected photochemical processes in green stem cells and the production of assimilates. These assumptions are related to observed changes in chlorophyll fluorescence parameters.

### Light-mediated regulation of nitrogen uptake dynamics

In our experiments, stem darkening decreased nitrogen uptake from soil in both NO_3_^–^ and NH_4_^+^ forms. Although NO_2_^–^ form is considered as toxic to plant cells [60], it was taken up more intensively by DP compared with NDP.

Plants prefer to take up nitrogen in the form of NO_3_^–^. Thus, the observed reduction in NO₃⁻ uptake under darkening reflects physiological adaptation to darkening, resulting in energy saving for NO_3_^–^ reduction in roots [61]. This suggests that light access to the stem is an important factor in the undisturbed process of nitrogen uptake and management in the plant.

Similar observations were reported in *Triticum aestivum* L. cv. ‘EM18’ [62]. Shoot illumination stimulated nitrate uptake by roots, whereas darkness reduced it. The authors suggest that this process is associated with the transport of sugars through the phloem.

In the present study, we suggest that, in DP plants with lower capacity to reduce NO_3_^–^ within stem chloroplasts, the uptake of NO_2_^–^ increases with increasing nitrogen demand. However, this interpretation requires further verification.

### Stable isotopes and C: N stoichiometry under darkening

Although C: N ratios and ^13^C and ^15^N isotope compositions did not differ significantly between treatments, general trends were observed. C: N ratios were higher in DP, indicating lower nitrogen content, especially in stems and leaves. Importantly, at the beginning of the experiment, DP stems were not yet darkened, and all plants were initially treated in a similar way, which may have influenced the final isotope and stoichiometric signatures, potentially reducing the contrast between treatments, especially in stems and roots.

The composition of ^13^C reflects carbon fixation and allocation in plants under physiologically stable conditions. The highest ^13^C values were observed in roots, whereas leaves showed the lowest values, with no clear differences between NDP and DP plants. This pattern is consistent with preferential discrimination of the lighter ¹²C isotope during photosynthesis via Rubisco, resulting in enrichment of ¹³C in transported assimilates allocated to stems and roots [63, 64].

The composition of δ^15^N is related more to nitrogen uptake, transport, and internal redistribution rather than total nitrogen content. The lowest δ^15^N values in both NDP and DP indicate that the roots are the primary site of nitrogen uptake. Differences between organs reflect internal allocation of nitrogen and metabolic demand. In stems, where δ^15^N is the highest, this may reflect nitrogen processing or temporary storage rather than immediate metabolic utilisation.

Differences in the C: N ratio, as well as δ^15^N, may reflect the different functions of the organs, with roots as nitrogen assimilating organs, while stems and leaves function as major sites of nitrogen assimilation. The roots assimilate nitrogen, but do not use it intensively in metabolic reactions, and nitrogen is transported to the aboveground parts of plants; therefore, the C: N ratio is high [63–65].

Lower δ^15^N values in NDP plants compared with DP may indicate stronger discrimination of the ^15^N isotope, potentially reflecting greater metabolic energy in the form of ATP and NADPH for selective nitrogen uptake, assimilation, and redistribution in plants. This process involves key enzymes – nitrate reductase (NR), responsible for catalysing the reaction of reducing nitrates to nitrites, nitrite reductase (NiR) active in the reaction of reduction of nitrite ions into ammonium ions in leaf chloroplasts or root plastids and glutamine synthetase (GS), which catalyses the reactions of glutamine biosynthesis involving ammonia. These enzymes prefer to fix the lighter nitrogen isotope ^14^N when available, which influences fractionation during its uptake and assimilation [65, 67]. Light availability is known to regulate NR and NiR activity, and their activity is reduced under dark conditions [68, 69].

Consistent with this, lower C: N ratios in DP roots suggest reduced carbon availability and transport to aboveground organs. In contrast, changes in stems and leaves may reflect reduced nitrogen assimilation and decreased redistribution under limited light conditions.

These observations are consistent with the results obtained for *Plantago lanceolata* L. growing in the difficult conditions of the desert-like environment and in the nearby forest. Demanding environmental conditions influenced the change in nitrogen uptake and the redistribution of various forms of nitrogen in the plant [70]. It is another piece of evidence that the lack of energy leads to limited nitrogen uptake and transport.

Overall, under limited light conditions, plants may adjust nitrogen metabolism to reduce energy demand, as nitrate reduction requires reducing power (ATP and NADPH) linked to the photosynthetic electron transport chain, rather than preferentially absorbing NO_2_^–^ as a nitrogen source [65, 71]. Although only stem light availability was manipulated, the resulting disruption in nitrogen metabolism suggests that photochemical processes in stem chloroplasts may contribute to systemic regulation of plant nitrogen economy and, consequently, root development and whole-plant growth.

To sum up, limited light access to tomato stems reduces photochemical processes and likely photosynthesis, which may affect assimilate redistribution between stems and roots. Low rETR values in stems and pith, regardless of treatment (NDP or DP), suggest a limited role of chloroplasts in photosynthesis in these tissues. However, despite this limited contribution to net carbon assimilation, stem chloroplasts may perform additional regulatory or metabolic functions, as indicated by ultrastructural observations and light-dependent physiological responses. Stem darkening affected chloroplast function and was associated with reduced nitrogen uptake and lower root biomass in DP plants.

## Materials and methods

### Plant cultivation

**Fig. 8:**
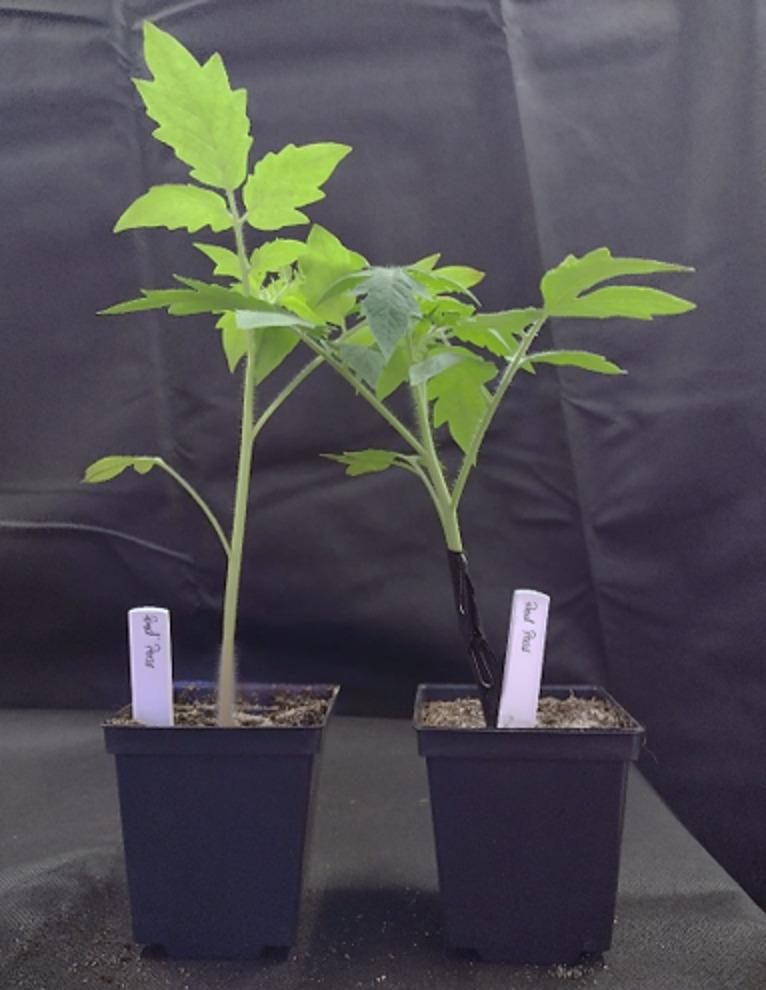
Plants with non-darkened (left) and darkened stems (right), prepared for further analyses; scale bar: 10 cm

The ‘Red Pear’ tomato (*Solanum lycopersicum* L.) plants were obtained from commercially purchased seeds (W. Legutko, Jutrosin, Poland). The seeds were germinated under natural light for 10 days using a previously sifted, market-available, universal soil substrate mixed with market-available sand at a 2:1 ratio. After 10 days, the seedlings were transferred to the individual 9 × 9 × 10 pots filled with the same substrate and placed in a growth chamber under controlled conditions: approximately 50% relative humidity, a 22/17°C day/night thermoperiod, and a 14/10 h day/night photoperiod. The light period started at 08:00, and the measurements were conducted during the light phase under stable illumination conditions. Illumination was provided by light-emitting diode (LED) lamps (PX759 Experimental LED Lamp, PXM, Podłęże, Poland) with the following spectral composition: 430 nm – 35 µmol m^−2^ s^−1^, 460 nm – 45 µmol m^−2^ s^−1^, 540 nm – 10 µmol m^−2^ s^−1^, 630 nm – 45 µmol m^−2^ s^−1^, 660 nm – 32 µmol m^−2^ s^−1^, 730 nm – 3 µmol m^−2^ s^−1^, resulting in the total active photon flux density of 170 µmol m^−2^ s^−1^. The growing seedlings were divided into two groups of 15 plants each: one with non-darkened stems (NDP – non-darkened plants) and one with stems darkened with a doubled agrotextile (DP – darkened plants), reducing light exposure by about 99% [72]. The agrotextile used for stem darkening is a nonwoven polypropylene material applied in agricultural systems, that enables gas exchange while primarily reducing light penetration [73]. As the plants increased in height, the stems of the plants in the second group were regularly darkened using additional parts of agrotextile to maintain near-dark conditions around the growing stems. The double layer ensured complete exclusion of PAR and eliminated potential lateral light leakage. All plants were watered simultaneously with a similar volume of tap water. For 8-week-old tomato plants (Fig. 8 and S1), the measurements described below were taken, and all plants were subsequently harvested for further analysis. The same plants were used for gas-exchange, fluorescence and biometric analysis. For Chl*a* fluorescence analyses, the pith was considered as a separate tissue, while the term ‘stem’ refers to the remaining stem tissues excluding the pith.

### Biometric analysis

For the determination of fresh and dry weights of roots and shoots (stems with leaves), plants were removed from the soil and gently rinsed with tap water to remove any remaining growing substrate. The shoot part of the tomato plants was then separated from the root parts at the level of the growing substrate. Subsequently, all organs were blotted dry with paper towels after rinsing and weighed to determine their fresh weight. To determine dry mass, the plant organs were dried for 24 h in an oven at 105 °C.

### Fluorescence of chlorophyll *a* measurement

Chlorophyll *a* fluorescence imaging was performed using a pulse-amplitude modulated (PAM) system (Imaging PAM, Walz, Effeltrich, Germany) equipped with a bank of blue LEDs (λ = 450 nm) and a charged-coupled device (CCD) camera for capturing fluorescence images. Measurements were conducted between 07:00 and 11:00 a.m. on leaves and stems dark-adapted for 30 min. Completely dark-adapted segments of *S. lycopersicum* stems were excised and immediately placed in a container filled with tap water to avoid tissue desiccation. The stems were then split with a razor blade and placed into a transparent polyethene (9 × 13 cm) sealable bag filled with water. Two parts of the same stem were positioned so that one part was illuminated from the outer side and the other from the inner tissues.

The minimal fluorescence level (F_0_), with all PSII reaction centres open, was measured using low-intensity (0.01 µmol m⁻^2^ s⁻^1^ of PAR) modulated blue measuring light (450 nm). Leaves and stems were then exposed to a saturating light pulse (ca. 2700 µmol m⁻^2^ s⁻^1^ PAR) to determine the maximum fluorescence yield with all PSII reaction centres closed (F_m_). Subsequently, leaves and stems were illuminated with blue actinic light (145 µmol m⁻^2^ s⁻^1^ PAR for leaves and 9 µmol m⁻^2^ s⁻^1^ PAR for stems) to match the photon flux density of the growth light. After approximately 4 min, the steady-state fluorescence (F_s_) was recorded, followed by the determination of the maximal fluorescence in the light-adapted state (F_m_). Different actinic light intensities were applied to leaves and stems to avoid overexcitation of stem tissues, which exhibit substantially lower photosynthetic capacity. The actinic light intensities used in PAM measurements were selected to reflect the respective growth light conditions and to ensure appropriate excitation without over-saturating stem tissues.

The maximum PSII photochemical efficiency was calculated as:

$$F_{v}/F_{m}=\frac{\left(F_{m}-F_{0}\right)}{F_{m}}$$

where F_v_ - variable fluorescence (F_v_ = F_m_ − F_0_), F_m_ - as described above, F_0_ - minimal fluorescence in the dark [47].

Raw F_0_ and F_m_ values used for F_v_/F_m_ calculation are provided in Supplementary Table S1.

The effective quantum yield of PSII photochemistry [Y(II)] was calculated as:

$$Y\left(II\right)=\frac{\left(F_{m}{'}-F_{s}\right)}{F_{m}{'}}$$

where F_m_’ - maximum fluorescence in light (after saturation pulse), F_s_ – steady-state fluorescence in light [74, 75].

The relative electron transport rate (ETR) was calculated as:

$$rETR=Y\left(II\right)\times{PAR}\times0.5\times{A}$$

where 0.5 – coefficient from the assumption that half of the energy goes to PSII, A - absorption of the measured surface, set to 0.84 for leaves and stems [46, 50].

The quantum yield of regulated energy dissipation in PSII [Y(NPQ)] was calculated as [74, 75]:

$$Y\left(NPQ\right)=\frac{F_{s}}{F_{m}{'}}-\frac{F_{s}}{F_{m}}$$

The non-regulated energy dissipation in PSII (Y(NO)) was calculated as [74]:

$$Y\left(NO\right)=\frac{{F}_{s}}{{F}_{m}}$$

The photochemical quenching (qP) was calculated as:

$$qP=\frac{\left(F_{m}{'}-F_{s}\right)}{\left(F_{m}{'}-F_{0}{'}\right)}$$

where F_0_’ - minimal fluorescence in light (estimated) [74, 75].

### Analysis of stem cross-section

Stem cross-sections were analysed for chlorophyll and lignin distribution based on autofluorescence, following the procedure described previously [13, 38]. Cross-sections of stems were prepared manually using a razor blade and examined in distilled water under a Nikon ECLIPSE Ni light and epifluorescence microscope (Nikon, Japan) equipped with a Digital Sight DS-Fi1c camera and NIS-Elements imaging software (version 4.11; Nikon, Japan). Excitation was provided by a high-pressure mercury lamp (HBO), and appropriate filter sets were used to select the desired excitation wavelengths. Lignin was detected using UV excitation (Ex 340–380 nm) and emission collected at 420–550 nm. Chlorophyll autofluorescence was recorded using blue light excitation (Ex 450–490 nm) and emission detected at 650–750 nm.

### Gas exchange measurement

Measurements of gas exchange were conducted under laboratory conditions using a Portable Photosynthesis System LI-6400XT (LI-COR Inc., Lincoln, NE, USA) with stems. Gas (CO_2_ and H_2_O) efflux rates were measured using a standard chamber equipped with a 6400-02B LED source. Each measurement consisted of four replicates, with one stem per replicate. The leaf cuvette conditions were maintained at a relative air humidity of 25%, air temperature in the cuvette set to a constant 26 °C, 400 µmol mol^–1^ of external CO_2_ concentration and a gas flow rate of 500 ± 2 µmol s^−1^. Measurements were performed at two levels of PAR: darkness (0 µmol m⁻^2^ s⁻^1^) to assess respiratory gas exchange and light (1500 µmol m⁻^2^ s⁻^1^) to determine light-saturated photosynthetic gas exchange. Gas exchange parameters were recorded for approximately 300 s per replicate until a stable reading was obtained.

The values of the stem conductance (g_*stem*_) have been calculated according to the formula [74]:

$$\:gstem=\frac{E\left(1000-\frac{W\mathrm{c}+W\mathrm{s}}{2}\right)}{W\mathrm{c}-W\mathrm{s}}$$

Where E is transpiration, W_c_ and W_s_ are the molar concentration of water vapour within the stem cortex and in the air sample, respectively.

The efflux of CO_2_ (E_CO2_) was calculated as follows [76]:

$$\:ECO_\mathrm{2}=\frac{F\left(C\mathrm{r}-C\mathrm{s}\left(\frac{1000-W\mathrm{r}}{1000-W\mathrm{s}}\right)\right)}{100}\times\:(-1)$$

where *F* is the air flow rate, *C*_*r*_ and *C*_*s*_ are reference and sample CO_2_ concentrations, W_r_ is the molar concentration of water vapour in reference air, and W_s_ is the molar concentration of water vapour within the stem.

### Chloroplast ultrastructure analysis

For Transmission Electron Microscopy (TEM) analysis of chloroplasts’ ultrastructure, cross-sections were collected from the midpoint of the stem region covered by agrotextile and from the corresponding stem height in plants grown without darkening. The samples were fixed for 2 h at room temperature in Karnovsky’s fixative (2% paraformaldehyde and 2.5% glutaraldehyde in 0.1 M sodium cacodylate buffer, pH 7.3) and subjected to a routine preparation procedure for epoxy resin embedding as described previously [77]. Semi-thin Sect.  (3 μm) stained with 0.1% toluidine blue were analysed under a light microscope (Olympus-Provis, Japan), while ultrathin (80 nm) sections collected on formvar-coated grids were examined under TEM (FEI 268D Morgagni, FEI Comp., Hillsboro, OR, USA) equipped with a 10 MPix Olympus-SIS ‘Morada’ digital camera (Olympus-SIS, Münster, Germany).

### The content of nitrogen forms in the soil

Analyses of soil total nitrogen and its forms content were performed in the Laboratory of Ecochemistry and Environmental Engineering, W. Szafer Institute of Botany Polish Academy of Sciences. Soil samples were sifted firstly through a sieve (2 mm mesh) and then shaken in water for 1 h. Total nitrogen content was determined using the Kjeldahl method using Kjeltec 8400 Auto Distillation (FOSS Analytics, Denmark) [78]. The analysis of N-NH_4_ form content was performed using an ion chromatograph Dionex DX-100 with Dionex IonPac™ CS12A column (Dionex, Sunnyvale, CA, USA), and a Dionex ICS-1100 ion chromatograph equipped with Dionex IonPac™ AS9-HC (Dionex) was applied for N-NO_2_ and N-NO_3_ content forms analysis.

### Analysis of the total carbon and nitrogen content and ^13^C, ^15^N discrimination

For analysis of total carbon and nitrogen content, as well as ^13^C and ^15^N discrimination, leaf, stem, and root tissues after drying (24 h at 105 °C in an oven) were thoroughly ground in a mortar to obtain a fine powder. Each sample was weighed separately and packed into specialised tin capsules (Elemental Microanalysis Ltd, Okehampton, UK), which were then placed into 96-well plates. Sample weights varied among plant organs, ranging from 3.5 to 4.5 mg for leaves, 3.5 to 4.1 mg for stems, and 3.0 to 5.0 mg for roots. Analyses of total carbon and nitrogen content, as well as ^13^C and ^15^N discrimination, were carried out at the Stable Isotope Facility (SIF), University of California (Davis, CA, USA), using an elemental analyser coupled to a continuous flow isotope ratio mass spectrometer (EA-IRMS) system (Elementar Analysensysteme GmbH, Langenselbold, Germany). Following combustion of the tissue samples at 950 °C and removal of residual oxygen and nitrogen oxides by passing the products over reduced copper. Carbon dioxide (CO_2_) and nitrogen (N_2_) were subsequently separated using a column in a gas chromatography method and carried for measurement to the IRMS. Enriched alanine, caffeine, glutamic acid, glutathione, and nylon were used as quality assurance reference materials, while scallop and amaranth flour were employed as quality control reference materials. Final results for carbon isotope composition - δ^13^C calculated as follows [79]:

$$\:{\:{\updelta\:}}^{13}C=\left(\frac{\frac{{}_{}{}^{13}C}{{}_{}{}^{12}C}sample}{\frac{{}_{}{}^{13}C}{{}_{}{}^{12}C}standard}-1\right)\times\:\:1000\:$$

and nitrogen isotope composition calculated as follows [80]:

$$\:{\:{\updelta\:}}^{15}N=\left(\frac{\frac{{}_{}{}^{15}N}{{}_{}{}^{14}N}sample}{\frac{{}_{}{}^{15}N}{{}_{}{}^{14}N}standard}-1\right)\times\:\:1000\:$$

were expressed relative to international standards: Vienna Pee Dee Belemnite (VPDB) for carbon and air for nitrogen [81].

### Statistical analysis

The obtained results have been analysed using the STATISTICA 13 package (StatSoft, Tulsa, OK, USA). The significance of differences in organ length and dry mass, photochemical parameters, gas exchange parameters, various nitrogen contents, isotope ^13^C discrimination rate and isotope ^15^N content between plants with non-darkened and darkened stems was assessed using a *t*-test at *p* ≤ 0.05 or Welch’s *t*-test for unequal variances and the Mann-Whitney test in case of non-parametric data, conducted separately for each organ or soil group. Mean values of the measured parameters are presented together with their standard deviation values.

## Conclusions

This study provides information about the role of photochemical processes taking place in tomato stems and their involvement in root system development. Although the photochemical activity of darkened stems is partially taken over by the leaves, limiting access of light to these parts of the plant limits the growth and development of the root system, but does not appear to have a detectable negative effect on the above-ground parts of the tomato under the conditions of this study. Restricting light exposure to the stems likely reduces energy production in the form of energy carriers, such as ATP and NADPH, resulting in decreased assimilate transport to the root. This disrupts the plant’s nitrogen uptake and balance, and the efficiency of photosynthesis in the stems, which in turn limits root system development. Moreover, it can be assumed that the chloroplasts present in tomato stems differ structurally and, as a consequence, functionally from those in leaves, which are specialised for efficient photosynthesis. However, this requires further research.

Because a well-developed root system is an important factor affecting plant growth and yield, the obtained results may suggest the possibility of agronomic practices aimed at increasing the efficiency of photochemical processes and photosynthesis in the stems, and consequently improving yields.

## Supplementary Information

Supplementary Material 1.

Supplementary Material 2.

## Data Availability

The dataset is prepared in Mendeley Data with assigned and reserved DOI. Now, the dataset is as a draft and will be published after online version of this publication.

## Abbreviations

δ^13^C: composition of carbon isotope
δ^15^N: composition of nitrogen isotope
CAM: Crassulacean Acid Metabolism
CCD: charged-coupled device
Chla: chlorophyll a
DP: darkened plants
EA-IRMS: elemental analyser coupled to a continuous flow isotope ratio mass spectrometer
ECO_2_: efflux of CO_2_
rETR: relative electron transport rate
F_0_: minimal fluorescence level
F_0_’: minimal fluorescence in light (estimated)
F_s_: steady-state fluorescence
F_m_: light-adapted state of fluorescence
F_v_: variable fluorescence
F_v_/F_m_: maximum quantum yield of photosystem II
GS: glutamine synthetase
g_stem_: stem conductance
LED: light-emitting diode
NDP: non-darkened plants
NiR: nitrite reductase
NPQ: non-photochemical quenching
NR: nitrate reductase
PAR: photosynthetically active radiation
PSII: photosystem II
rETR: relative electron transport rate
TEM: transmission electron microscopy
qP: photochemical quenching
VPDB: Vienna Pee Dee Belemnite
Y(II): quantum yield of PSII photochemistry
Y(NO): non-regulated energy dissipation in PSII
Y(NPQ): quantum yield of regulated energy dissipation in PSII
