# Supplementary material for: Beyond the leaves: functional role of chlorophyllous stems in tomato (Solanum lycopersicum L.) and their impact on nitrogen balance and root development
Source: BMC Plant Biol. 2026 May 19;26:1184. doi: 10.1186/s12870-026-08992-y (PMC13361740; doi:10.1186/s12870-026-08992-y)
Supplement: Supplementary file 1 — Supplementary Material 1. [file 12870_2026_8992_MOESM1_ESM.docx]

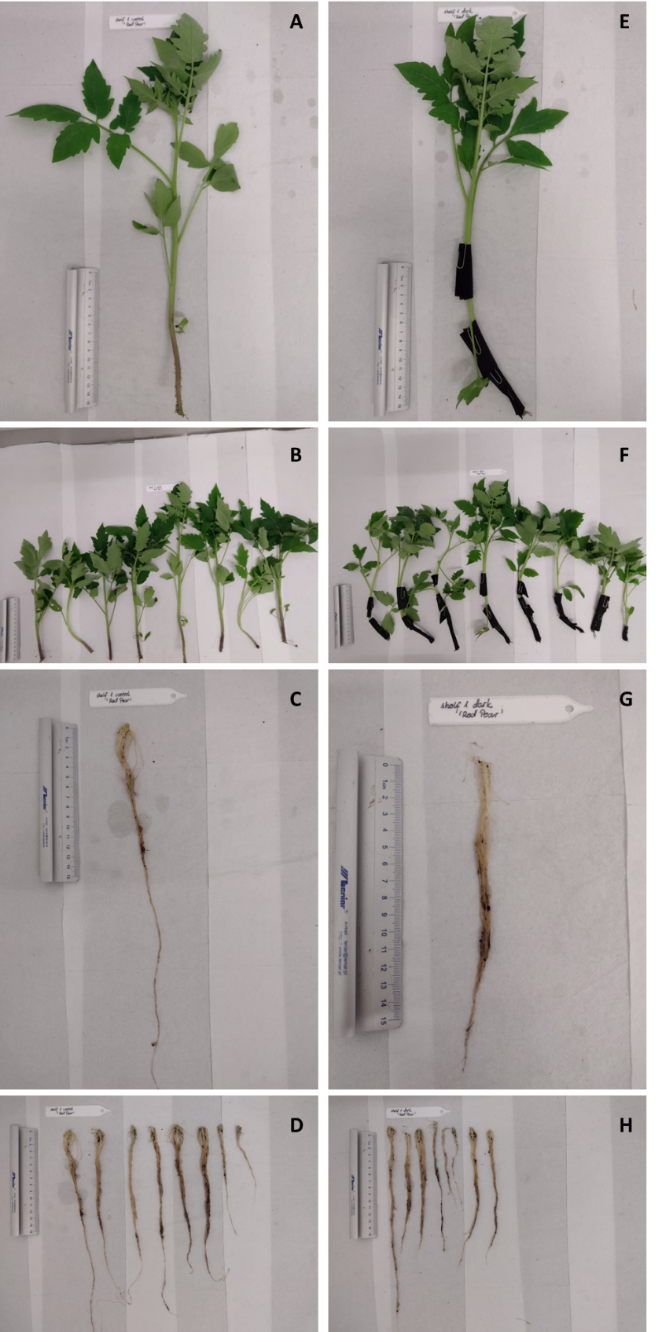


**Figure S1**

8-week-old ‘Red Pear’ tomato plants after harvest. Aboveground parts (A, B) and roots (C, D) of plants with non-darkened stems during growth, and aboveground parts (E, F) and roots (G, H) of plants with darkened stems during growth
