# Supplementary material for: Beyond the leaves: functional role of chlorophyllous stems in tomato (Solanum lycopersicum L.) and their impact on nitrogen balance and root development
Source: BMC Plant Biol. 2026 May 19;26:1184. doi: 10.1186/s12870-026-08992-y (PMC13361740; doi:10.1186/s12870-026-08992-y)
Supplement: Supplementary file 2 — Supplementary Material 2. [file 12870_2026_8992_MOESM2_ESM.docx]

**Table S1**

The raw data of minimal fluorescence in the dark (F_0_) and maximal fluorescence in the light-adapted state (F_m_) of leaves, stems, and piths of 8-week-old ‘Red Pear’ tomato (*Solanum lycopersicum* L.) plants with non-darkened (NDP) and darkened stems (DP). The term ‘stem’ refers to all stem tissues excluding the pith.
